# Effect of Saxagliptin on Endothelial Function in Patients with Type 2 Diabetes: A Prospective Multicenter Study

**DOI:** 10.1038/s41598-019-46726-3

**Published:** 2019-07-15

**Authors:** Masato Kajikawa, Tatsuya Maruhashi, Takayuki Hidaka, Shogo Matsui, Haruki Hashimoto, Yuji Takaeko, Yukiko Nakano, Satoshi Kurisu, Yasuki Kihara, Farina Mohamad Yusoff, Shinji Kishimoto, Kazuaki Chayama, Chikara Goto, Kensuke Noma, Ayumu Nakashima, Takafumi Hiro, Atsushi Hirayama, Kazuki Shiina, Hirofumi Tomiyama, Shusuke Yagi, Rie Amano, Hirotsugu Yamada, Masataka Sata, Yukihito Higashi

**Affiliations:** 1 Division of Regeneration and Medicine, Medical Center for Translational and Clinical Research, Hiroshima University Hospital, Hiroshima, Japan; 2 Department of Cardiovascular Medicine, Graduate School of Biomedical and Health Sciences, Hiroshima University, Hiroshima, Japan; 3 Department of Cardiovascular Regeneration and Medicine, Research Institute for Radiation Biology and Medicine, Hiroshima University, Hiroshima, Japan; 4 Department of Gastroenterology and Metabolism, Institute of Biomedical and Health Sciences, Graduate School of Biomedical and Health Sciences, Hiroshima University, Hiroshima, Japan; 5 Department of Physical Therapy, Hiroshima International University, Hiroshima, Japan; 6 Division of Cardiology, Department of Medicine, Nihon University School of Medicine, Tokyo, Japan; 7 Department of Cardiology, Tokyo Medical University, Tokyo, Japan; 8 Department of Cardiovascular Medicine, Institute of Health Biosciences, The University of Tokushima Graduate School, Tokushima, Japan

**Keywords:** Interventional cardiology, Outcomes research

## Abstract

The dipeptidyl peptidase-4 inhibitor saxagliptin is a widely used antihyperglycemic agent in patients with type 2 diabetes. The purpose of this study was to evaluate the effects of saxagliptin on endothelial function in patients with type 2 diabetes. This was a prospective, multicenter, interventional study. A total of 34 patients with type 2 diabetes were enrolled at four university hospitals in Japan. Treatment of patients was initially started with saxagliptin at a dose of 5 mg daily. Assessment of endothelial function assessed by flow-mediated vasodilation (FMD) and measurement of stromal cell-derived factor-1α (SDF-1α) were conducted at baseline and at 3 months after treatment with saxagliptin. A total of 31 patients with type 2 diabetes were included in the analysis. Saxagliptin significantly increased FMD from 3.1 ± 3.1% to 4.2 ± 2.4% (P = 0.032) and significantly decreased total cholesterol from 190 ± 24 mg/dL to 181 ± 25 mg/dL (P = 0.002), glucose from 160 ± 53 mg/dL to 133 ± 25 mg/dL (P < 0.001), HbA1c from 7.5 ± 0.6% to 7.0 ± 0.6% (P < 0.001), urine albumin-to-creatinine ratio from 63.8 ± 134.2 mg/g to 40.9 ± 83.0 mg/g (P = 0.043), and total SDF-1α from 2108 ± 243 pg/mL to 1284 ± 345 pg/mL (P < 0.001). These findings suggest that saxagliptin is effective for improving endothelial function.

## Introduction

Endothelial dysfunction occurs in the early stage of atherosclerosis and plays a key role in the progression of atherosclerosis^1,2^. Measurements of flow-mediated vasodilation (FMD), which is an index of endothelium-dependent vasodilation, have frequently been utilized to evaluate endothelial function^3–6^. Endothelial dysfunction is an independent predictor of vascular events^7–10^. Type 2 diabetes is associated with endothelial dysfunction and is a risk factor for systemic atherosclerosis and cardiovascular events^11–14^. Hyperglycemia in diabetes induces oxidative stress, which is a trigger of endothelial dysfunction by reducing nitric oxide (NO) bioavailability^13,14^. Therefore, it is necessary to identify interventions that can prevent endothelial dysfunction in patients with type 2 diabetes.

Dipeptidyl peptidase-4 (DPP-4) inhibitors are widely used antihyperglycemic agents in patients with type 2 diabetes^15–17^. It has been demonstrated that DPP-4 inhibition has vascular protective benefits via the regulation of several substrate factor activities^18^. Stromal cell-derived factor-1α (SDF-1α), one of the DPP-4 substrates, participates in the repair of vascular injury by mobilization of endothelial progenitor cells^19,20^. Several experimental studies have shown that a DPP-4 inhibitor has a beneficial effect on the endothelial function through increasing SDF-1α levels^21,22^. However, there is no information on the effects of saxagliptin on SDF-1α in humans.

The relationship between treatment with saxagliptin and endothelial function in patients with type 2 diabetes has been reported^23,24^. However, previous studies were single center studies with a limited number of patients. Therefore, we conducted a prospective, multicenter study to evaluate the effects of saxagliptin on endothelial function and circulating SDF-1α levels in patients with type 2 diabetes.

## Results

### Clinical characteristics

We enrolled 34 patients with type 2 diabetes. Three patients including 1 patient who discontinued the intervention and 2 patients who had a protocol deviation were excluded from the analysis. The baseline clinical characteristics of the 31 patients before and after treatment with saxagliptin are summarized in Table 1. The 31 patients included 22 men (71.0%) and 9 women (29.0%), and 29 (93.5%) of the patients had hypertension, 23 (74.2%) had dyslipidemia, 18 (58.1%) had a history of smoking, 10 (32.3%) had history of coronary artery disease, and 2 (6.5%) had a history of stroke.

**Table 1:** Patient characteristics and changes in parameters before and after treatment.

Variables	Baseline n = 31	12 weeks n = 31	P value
Age, yr	64 ± 13		
Gender, men/women	22/9		
Body mass index, kg/m^2^	27.8 ± 5.6	27.7 ± 5.9	0.354
Body weight, kg	75.8 ± 19.2	75.4 ± 20.7	0.341
Systolic blood pressure, mmHg	126 ± 17	126 ± 17	0.877
Diastolic blood pressure, mmHg	78 ± 8	76 ± 9	0.473
eGFR, mL/min/1.73 m^2^	71.2 ± 16.5	70.2 ± 14.9	0.162
Total cholesterol, mg/dL	190 ± 24	181 ± 25	0.002
Triglycerides, mg/dL	175 ± 167	144 ± 74	0.247
HDL cholesterol, mg/dL	55 ± 18	53 ± 17	0.300
LDL cholesterol, mg/dL	105 ± 26	99 ± 24	0.208
Glucose, mg/dL	160 ± 53	133 ± 25	< 0.001
HbA1c, (%)	7.5 ± 0.6	7.0 ± 0.6	< 0.001
ACR, (mg/g)	63.8 ± 134.2	40.9 ± 83.0	0.043
Medical history, n (%)			
Diabetes duration, years	7.9 ± 10.3		
Hypertension, n (%)	29 (93.5)		
Dyslipidemia, n (%)	23 (74.2)		
Previous coronary heart disease, n (%)	10 (32.3)		
Previous stroke, n (%)	2 (6.5)		
Current smoker, n (%)	5 (16.1)		
Former smoker, n (%)	18 (58.1)		
Medications, n (%)			
Calcium-channel blockers, n (%)	18 (58.1)	18 (58.1)	NA
Renin angiotensin system inhibitors, n (%)	22 (71.0)	22 (71.0)	NA
Statins, n (%)	17 (54.8)	17 (54.8)	NA
Biguanides, n (%)	7 (22.6)	7 (22.6)	NA
Sulfonylurea, n (%)	3 (9.7)	3 (9.7)	NA
Thiazolidinedione, n (%)	0 (0.0)	0 (0.0)	NA
Alpha-glucosidase inhibitors, n (%)	3 (9.7)	3 (9.7)	NA
SGLT-2 inhibitors, n (%)	7 (22.6)	7 (22.6)	NA
Insulin, n (%)	0 (0.0)	0 (0.0)	NA

Results are presented as mean ± SD for continuous variables and percentages for categorical variables.

eGFR indicates estimated glomerular filtration rate; HDL, high-density lipoprotein; LDL, low-density lipoprotein; ACR, albumin-to-creatinine ratio; SGLT-2, sodium glucose cotransporter-2; NA, not applicable.

Changes in parameters after treatment were evaluated using paired t test.

### Effects of saxagliptin on endothelial function and parameters

**Figure 1:**
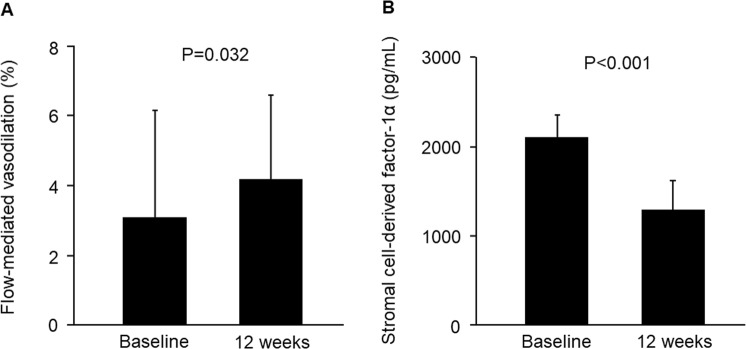
Bar graphs show flow-mediated vasodilation (**A**) and stromal cell-derived factor-1α (**B**) before the beginning of treatment and after 12 weeks of treatment.

Saxagliptin significantly increased FMD from 3.1 ± 3.1% to 4.2 ± 2.4% (P = 0.032, Fig. 1A). Saxagliptin significantly decreased total cholesterol, glucose, HbA1c, urine albumin-to-creatinine ratio (ACR) (Table 1), and SDF-1α (from 2108 ± 243 pg/mL to 1284 ± 345 pg/mL, P < 0.001, Fig. 1B). There were no significant differences in body mass index, body weight, systolic blood pressure, diastolic blood pressure, eGFR, triglycerides, high-density lipoprotein cholesterol, and low-density lipoprotein cholesterol before and after 12 weeks of saxagliptin treatment. Changes in FMD did not correlate with changes in systolic blood pressure (r = 0.22, P = 0.36), changes in diastolic blood pressure (r = −0.15, P = 0.58), changes in glucose (r = 0.19, P = 0.32), changes in HbA1c (r = −0.08, P = 0.68), changes in ACR (r = 0.29, P = 0.11), or changes in SDF-1α (r = −0.03, P = 0.89).

### Adverse effects

None of the patients withdrew from the study because of adverse effects associated with the treatment. One patient reported mild constipation. Two patients had mild liver enzyme elevation. One patient reported bone fracture after an incidental fall. There were no hypoglycemic events during the study period.

## Discussion

This study was a prospective, multicenter, interventional study to evaluate the effects of saxagliptin on endothelial function in patients with type 2 diabetes. Treatment with saxagliptin significantly increased FMD and significantly decreased SDF-1α and ACR.

We showed that saxagliptin significantly improved endothelial function. Several potential mechanisms by which saxagliptin improves endothelial function has been proposed. It is well known that DPP-4 inhibitors enhance systemic and tissue glucagon-like peptide-1 (GLP-1) levels^18,25^. Previous studies showed that GLP-1 per se directly enhances phosphorylation of adenosine monophosphate-activated protein kinase (AMPK) and Akt in endothelial cells^26,27^. DPP-4 inhibitor-mediated AMPK activation has been shown to improve endothelial function by counteracting oxidative stress in endothelial cells^25,26^. However, there is controversy about the effects of treatment with DPP-4 inhibitors on FMD^23,24,28^. Kitao *et al*. showed that FMD does not alter after administration of vildagliptin^28^. They enrolled type 2 diabetic patients treated with metformin and the mean value of baseline FMD was 5.48%. Nafisa *et al*. showed that metformin improves endothelial function in patients with diabetes mellitus^29^. It is thought that endothelial function was already improved by pretreatment with metformin.

SDF-1α increased by a DPP-4 inhibitor has been shown to enhance homing of endothelial progenitor cells and thereby exert vascular protection^19–22,25,30^. In the present study, a DPP-4 inhibitor significantly decreased total SDF-1α levels. Several clinical studies and the present study have shown that treatment with DPP-4 inhibitors significantly decreases the total amount of SDF-1α^31,32^. Lovshin *et al*. reported that administration of sitagliptin significantly increased intact SDF-1α and decreased truncated SDF-1α, resulting in an decrease in the total amount of SDF-1α^33^. The reason for this discrepancy between clinical observations and experimental studies is due to the methodological differences in SDF-1α assays. In addition, experimental studies have shown that a DPP-4 inhibitor significantly increased SDF-1α levels in a murine model of type 1 diabetes^34,35^. Further studies in which the relationship between effects of DPP-4 inhibitors on SDF-1α levels is evaluated in a murine model of type 2 diabetes may reveal the reason for this discrepancy.

Chronic kidney disease is one of the complications of type 2 diabetes mellitus. Urine albumin excretion (random urine ACR) is a marker for kidney damage, and increased ACR is a risk factor for end-stage renal disease (ESRD) and cardiovascular events^36,37^. Although angiotensin-converting enzyme inhibitors or angiotensin-receptor blockers are recommended to reduce the prevalence of ESRD in patients with diabetes, it is well known that patients with diabetes have a high residual risk of ESRD^38–40^. Several experimental studies have suggested that saxagliptin improves renal function^41,42^. Recently, a large clinical trial has shown that treatment with saxagliptin improved ACR compared with that in the placebo group after a median follow-up period of 2.1 years^43^. In the present study, we confirmed that 3-month treatment with saxagliptin significantly decreased ACR. However, the effects of saxagliptin on the risk of renal outcomes remains inconclusive^30^. Further studies with a longer duration are needed to evaluate the effects of saxagliptin on renal outcomes.

Several factors are known to affect vascular tone through NO metabolism in endothelial cells. The β_2_ adrenergic receptors and glucose metabolism are involved in the release of NO, leading to alteration in vasoconstriction and vasodilation of blood vessels^11,12,44^. In the present study, changes in FMD were not associated with changes in systolic blood pressure, changes in diastolic blood pressure, changes in glucose, changes in HbA1c, or changes in ACR, suggesting that saxagliptin improves endothelial function independently of its effects on glucose metabolism and renal function. In addition, there was no significant relationship between changes in FMD and changes in SDF-1α. However, there was not enough power to draw a negative conclusion. We cannot deny the possibility that saxagliptin improves endothelial function by improving glucose metabolism and renal function and by inducing an increase in SDF-1α-related endothelial progenitor cells. A large clinical trial is needed to confirm the factors that improve endothelial function in patients treated with saxagliptin.

The present study has some limitations. First, this was not a randomized and placebo-control study design and was a single-arm. In addition, the number of subjects was relatively small. However, it was clearly shown that saxagliptin improves endothelial function assessed by FMD in this prospective, multicenter study. In addition, the integrity of the data and the accuracy of the data analysis are ensured by regulatory authorities (independent data center, data monitoring committee, and audit team). Second, we evaluated only the 3-month effects of saxagliptin on endothelial function. Long-term interventions are needed to determine whether the 3-month effects of saxagliptin are sustained over time. Third, although measurements of reactive hyperemia index and endothelial progenitor cells as an index of endothelial function would enable more specific conclusions concerning the role of saxagliptin in endothelial function to be drawn, we cannot perform additional experiments to evaluate endothelial function. In the present study, measurement of FMD was performed by sonographers specialized in FMD measurement. To decrease the measurement variability of FMD, all of the sonographers received training for a standard protocol of FMD measurement at the core laboratory located in Tokyo Medical University. Previously, we confirmed that the FMD values measured at each hospital had a good correlation with the FMD values measured at a core laboratory (r = 0.838, P < 0.001)^45^. Finally, some antidiabetic agents such as metformin have been shown to improve endothelial function^29^. Of the 31 patients, 14 patients (45.2%) took antidiabetic agents. Although none of patients changed medications at any time throughout the study, we cannot deny the possibility that medications affected the results of this study.

In conclusion, treatment with saxagliptin is effective for improving endothelial function. Further studies are needed to assess the long-term effects of saxagliptin on vascular function, onset of cardiovascular disease, and cardiovascular events.

## Methods

### Study participants

Between June 2016 and June 2017, we enrolled 34 patients with type 2 diabetes at four university hospitals in Japan. Diabetes mellitus was defined according to the American Diabetes Association^46^. Estimated glomerular filtration rate (eGFR) was calculated by the following equation: 194 × serum creatinine^−1.094^ × age^−0.287^ (×0.739 if women)^47^. The inclusion criteria were as follows: (1) patients with type 2 diabetes, (2) age ≥20 years, and (3) HbA1c level ≥7.0% and <9.0%. The exclusion criteria were as follows: (1) treatment with DPP-4 inhibitors, GLP-1, or insulin, (2) a history of myocardial infarction or cerebrovascular disease within three months prior to the study, (3) a history of diabetic ketoacidosis or diabetic coma within three months prior to the study, (4) serious hepatic dysfunction, (5) eGFR < 50 mL/min per 1.73 m^2^, (6) pregnancy or possible pregnancy, and (7) a history of malignant disease within five years prior to the study. This study was approved by the ethical committee of Hiroshima University Graduate School of Medicine. The study was executed in accordance with the Good Clinical Practice guidelines. All patients gave written informed consent for participation in the study.

### Study protocol

This was a prospective, multicenter, interventional study. Treatment of patients was initially started with saxagliptin at a dose of 5 mg daily. Active treatment was then carried out for 12 weeks, and the time course of the effects of saxagliptin was evaluated.

The subjects were instructed not to eat, smoke, take caffeine and drink alcohol for about 12 hours before investigations. Data of investigations were obtained as each subject were put in the supine position in a quiet, dark, air-conditioned room (constant temperature of 22–25 °C). Venous blood samples were drawn from the left antecubital vein. FMD was measured after 30 minutes of resting in the supine position.

### Study management

Details of the organization of this study is as provided in the online-only Data Supplement (Supplementary Text). The independent data monitoring committee independently reviewed accrual, safety, and maturity of the data. The funding source had no role in study design or conduct, data collection, data management, analysis and interpretation of the data, and manuscript preparation. We abide with the relevant guidelines and regulations in performing the methods of this study.

### Measurement of FMD

FMD evaluation was performed using the high-resolution ultrasonography system (UNEXEF18G, UNEX Co, Nagoya, Japan). The protocol for measurement of FMD was as previously described^48^. In brief, the longitudinal images of the brachial artery were assessed at before and after a vascular response were generated by reactive hyperemia after a 5-min period of forearm occlusion. FMD was defined as the maximal percentage change in vessel diameter from the baseline value.

### Measurement of total SDF-1α level and urinary albumin and creatinine levels

SDF-1α was measured by using an enzyme-linked immunosorbent assay kit (Human CXCL12/SDF-1α immunoassay, R&D Systems, Minneapolis, USA). Urinary albumin and creatinine were measured in single voided urine samples, and the albumin-to-creatinine ratio (ACR) was calculated.

### Statistical analysis

For the present study, we estimated that 28 patients were needed with α = 0.05 and a power of 0.8 and with the expectation of at least 1.0% difference between the pre- and post-intervention values of FMD^49^. Finally, we enrolled 34 patients with consideration for 20% dropouts. Results are shown as the means ± SD for continuous variables and numbers (%) for categvorical variables. P < 0.05 was considered statistical significant. Changes in FMD and parameters before and after treatment with saxagliptin were evaluated using the paired t-test. Correlations between variables were performed by Pearson’s correlation analysis. The data were processed using the software package Stata version 9 (Stata Co., College Station, Texas, USA).

## Supplementary information

supplementary data
