# Supplementary material for: Effect of Saxagliptin on Endothelial Function in Patients with Type 2 Diabetes: A Prospective Multicenter Study
Source: Sci Rep. 2019 Jul 15;9:10206. doi: 10.1038/s41598-019-46726-3 (PMC6629702; doi:10.1038/s41598-019-46726-3)
Supplement: Supplementary file 1 — supplementary data [file 41598_2019_46726_MOESM1_ESM.doc]

**Online Supplement**

**Effect of Saxagliptin on Endothelial Function in Type 2 Diabetes: A Prospective Multicenter Study**

Brief title: Saxagliptin and endothelial function

Masato Kajikawa, MD, PhD;^1^ Tatsuya Maruhashi, MD, PhD;^2^ Takayuki Hidaka, MD, PhD;^2^ Shogo Matsui, MD;^2^ Haruki Hashimoto, MD;^2^ Yuji Takaeko, MD;^2^ Yukiko Nakano, MD, PhD;^2^ Satoshi Kurisu, MD, PhD;^2^ Yasuki Kihara, MD, PhD;^2^ Farina Mohamad Yusoff, MD;^3^ Shinji Kishimoto, MD, PhD;^3^ Kazuaki Chayama, MD, PhD;^4^ Chikara Goto, PhD;^5^ Kensuke Noma, MD, PhD;^1,3^ Ayumu Nakashima, MD, PhD;^3^ Hiro Takafumi, MD, PhD;^6^ Atsushi Hirayama, MD, PhD;^6^ Kazuki Shiina, MD, PhD;^7^ Hirofumi Tomiyama, MD, PhD, FAHA;^7^ Shusuke Yagi, MD, PhD;^8^ Rie Amano, RMS;^8^ Hirotsugu Yamada, MD, PhD; ^8^ Masataka Sata, MD, PhD;^8^

Yukihito Higashi, MD, PhD, FAHA^1,3^

^1^Division of Regeneration and Medicine, Medical Center for Translational and Clinical Research, Hiroshima University Hospital, Hiroshima, Japan

^2^Department of Cardiovascular Medicine, Graduate School of Biomedical and Health Sciences, Hiroshima University, Hiroshima, Japan

^3^Department of Cardiovascular Regeneration and Medicine, Research Institute for Radiation Biology and Medicine, Hiroshima University, Hiroshima, Japan

^4^Department of Gastroenterology and Metabolism, Institute of Biomedical and Health Sciences, Graduate School of Biomedical and Health Sciences, Hiroshima University Hiroshima, Japan

^5^Department of Physical Therapy, Hiroshima International University, Hiroshima, Japan

^6^ Division of Cardiology, Department of Medicine, Nihon University School of Medicine, Tokyo, Japan

^7^Department of Cardiology, Tokyo Medical University, Tokyo, Japan

^8^Department of Cardiovascular Medicine, Institute of Health Biosciences, The University of Tokushima Graduate School, Tokushima, Japan

Address for correspondence: Yukihito Higashi, MD, PhD, FAHA

Department of Cardiovascular Regeneration and Medicine,

Research Institute for Radiation Biology and Medicine (RIRBM), Hiroshima University

1-2-3 Kasumi, Minami-ku, Hiroshima 734-8551, Japan

Phone: +81-82-257-5831 Fax: +81-82-257-5831

E-mail: [yhigashi@hiroshima-u.ac.jp](mailto:yhigashi@hiroshima-u.ac.jp)

**Supplemental Text**

**Organization of this study**

1. **Chief Investigator**

Yukihito Higashi (Hiroshima University, Hiroshima, Japan).

1. **Participating Hospitals**

Yukihito Higashi (Hiroshima University, Hiroshima, Japan); Taishiro Chikamori (Tokyo Medical University, Tokyo, Japan); Atsushi Hirayama (Nihon University School of Medicine, Tokyo, Japan); Masataka Sata (University of Tokushima, Tokushima, Japan).

1. **Data Center**

Japan Academic Research Forum, Osaka, Japan

1. **Measurement Laboratories**

Centralized measurement laboratories for FMD: Yukihito Higashi (Hiroshima University, Hiroshima, Japan).

Measurement laboratories for blood and urine samples: SRL, Tokyo, Japan.

1. **Independent Data Monitoring Committee**

Ikumi Tominaga, Toshihide Oshima (Japan Academic Research Forum, Osaka, Japan).

1. **Independent Audit Team**

Masaaki Ozawa (Site Support Institute Corporation, Tokyo, Japan)

1. **Independent Statistical Analysis**

Increase Corporation, Tokyo, Japan
